# Point prevalence study of antibiotic appropriateness and possibility of early discharge from hospital among patients treated with antibiotics in a Swiss University Hospital

**DOI:** 10.1186/s13756-022-01104-z

**Published:** 2022-05-07

**Authors:** Estelle Moulin, Noémie Boillat-Blanco, Giorgio Zanetti, Catherine Plüss-Suard, Serge de Vallière, Laurence Senn

**Affiliations:** 1 Prevention and Infection Control Unit, Department of Medicine, Lausanne University Hospital (CHUV) and University of Lausanne, Chemin de Mont-Paisible 18, 1011 Lausanne, Switzerland; 2 Service of Infectious Diseases, Lausanne University Hospital and University of Lausanne, Lausanne, Switzerland; 3 Swiss Centre for Antibiotic Resistance (ANRESIS), Institute for Infectious Diseases, University of Bern, Bern, Switzerland

**Keywords:** Antibiotic stewardship programs, Outpatient parenteral therapy, Early discharge

## Abstract

**Background:**

The growing threat of multidrug resistant organisms have led to increasingly promote prudent and rational use of antimicrobials as well as early hospital discharge plan. Antibiotic stewardship programs (ASP) have been developed as multifaceted approaches to improve use of current antibiotics and are now widely applied through different strategies. Proactive interventions are still limited in Switzerland and data on antimicrobial appropriateness and early discharge strategies are lacking. We aimed to describe the opportunities of antibiotics prescriptions optimization at Lausanne University Hospital, Switzerland and evaluate the suitability for early discharge among patients receiving antibiotics. The need for outpatient medical structures was also assessed.

**Methods:**

We conducted a point prevalence survey of antibiotic prescriptions in adult medical and surgical units with exclusion of intermediate and intensive care units. All hospitalized patients receiving a systemic antibiotic on the day of evaluation were included. An infectious diseases specialist evaluated antimicrobial appropriateness and assessed suitability for discharge according to medical and nursing observations. The need of flexible additional outpatient facility for a close medical follow-up was evaluated concomitantly.

**Results:**

A total of 564 patients’ files were reviewed. 182 (32%) patients received one or more systemic antibiotic: 62 (34%) as a prophylaxis and 120 (66%) as a treatment with or without concomitant prophylaxis. 37/62 (60%) patients receiving prophylaxis had no indication to continue the antibacterial. Regarding the patients treated with antibiotics, 69/120 (58%) presented at least one opportunity for treatment optimization, mainly interruption of treatment. A previous ID consultation was recorded for 55/120 (46%) patients, of whom 21 (38%) could have benefited from antimicrobial therapy optimization on the day of the point assessment. 9.2% patients were eligible for discharge of whom 64% could leave the hospital with a close outpatient follow-up for infectious issues.

**Conclusions:**

This point prevalence study offers precious indicators for tailoring future antibiotic stewardship interventions that can be combined with early discharge. Any centre considering implementing ASP should conduct this type of analysis with a pragmatic approach to gain insight into local practices and needed resources.

## Introduction

To counter the threat of emergence and spread of multidrug resistant organisms, there is an increased focus on promoting prudent and rational use of antimicrobials as well as shortening hospital stays. In the literature, the proportion of inappropriate antibiotic courses in the hospital setting ranges between 9 and 64%, although difficult to compare because of different methods of assessment and different ways of reporting [1–7]. To further decrease the risk of nosocomial infections, selection and transmission of resistant bacteria, antibiotic optimization should be combined with an early discharge and outpatient care plan that could be included in an antibiotic stewardship program. Published studies enrolling patients with various infectious diseases in several European countries suggest that 10–50% of patients could be discharged earlier on an appropriate oral antibiotic therapy [8–10]. Moreover, evaluation by an infectious diseases team can help to identify patients who could be managed in the community with appropriate resources, including Outpatient parenteral antimicrobial therapy (OPAT) [2, 11, 12].

Antibiotic consumption in large Swiss hospitals (more than 500 beds) ranged in recent years between 55.4 and 60 Defined Daily Doses (DDDs) per 100 bed-days and is close to the median observed in the European Surveillance of Antimicrobial Consumption Network [13] The Swiss Federal Council adopted in 2015 an antibiotic resistance strategy (StAR for Swiss Antibiotic Resistance Strategy) [14] with the aim to define overarching objectives in many strategic fields of activity including appropriate use of antibiotics in human medicine. Nevertheless, proactive antibiotic stewardship interventions remain limited so far in Switzerland. Although unnecessary or inappropriate antimicrobial therapies were previously reported in Swiss acute care hospital settings, only limited data on appropriateness of antimicrobial prescriptions exist that could help to tailor future antimicrobial stewardship interventions [1, 3, 15]. In addition, there is a need to evaluate the requirement of adapted outpatient supports to shorten hospital stays.

This study aimed to assess the appropriateness of antibiotic use (indication, duration, route of administration, spectrum and dosing) in a Swiss University Hospital, the opportunity of early discharge, as well as the requirement for outpatient medical structures for close medical follow-up of the infectious status. The research hypothesis was that a systematic evaluation by a dedicated antibiotic stewardship team can contribute to improve antibiotic prescriptions as well as identify opportunities for early discharge with adapted monitoring and follow-up support in the community.

## Method

### Setting and study design

Lausanne University Hospital, Switzerland, is an 1100-acute somatic bed teaching hospital, with a role of primary and tertiary care covering all specialties. Infectious diseases (ID) consultations are available 7 days a week, 24 h a day. The ID team intervenes on request for specific cases, but also proactively give advice for severe infections, including bacteraemia, endocarditis and osteo-articular infections. Physicians have access to institutional guidelines on empirical antimicrobial therapies developed by ID specialists, microbiologists and pharmacists in collaboration with medical and surgical specialists. These guidelines are regularly updated, and are available online or as a pocket guide [16]. Additionally, an outpatient parenteral antibiotic therapy (OPAT) unit was created in 2014 and proposes consultations 5/7 days. An average of 330 outpatients per year are treated for a median of 15 days of intravenous antimicrobial treatment, corresponding to more than 4800 days of ambulatory parenteral treatment per year [17].

A dedicated ID specialist conducted a point prevalence study of antibiotic prescriptions and early discharge opportunities in all adult medical and surgical acute care units of the hospital over four months (March-June), with the exception of intermediate and intensive care units. Patients hospitalized in these wards were not included since eligibility for early discharge was not assessable. For each visited unit, the date of evaluation was scheduled in advance in order to have a nurse or a medical doctor available to answer questions if necessary.

All hospitalized patients receiving a systemic antibiotic prescribed as treatment or prophylaxis on the punctual assessment were included. They were identified using the electronic patient files (Soarian®) or the paper prescription records (electronic patient files were not available in all wards). Assessment of the possibility of early discharge was only evaluated among patients receiving antibiotics for treatment and not for prophylaxis.

### Assessment of the appropriateness of the antibiotic therapy

**Fig. 1:**
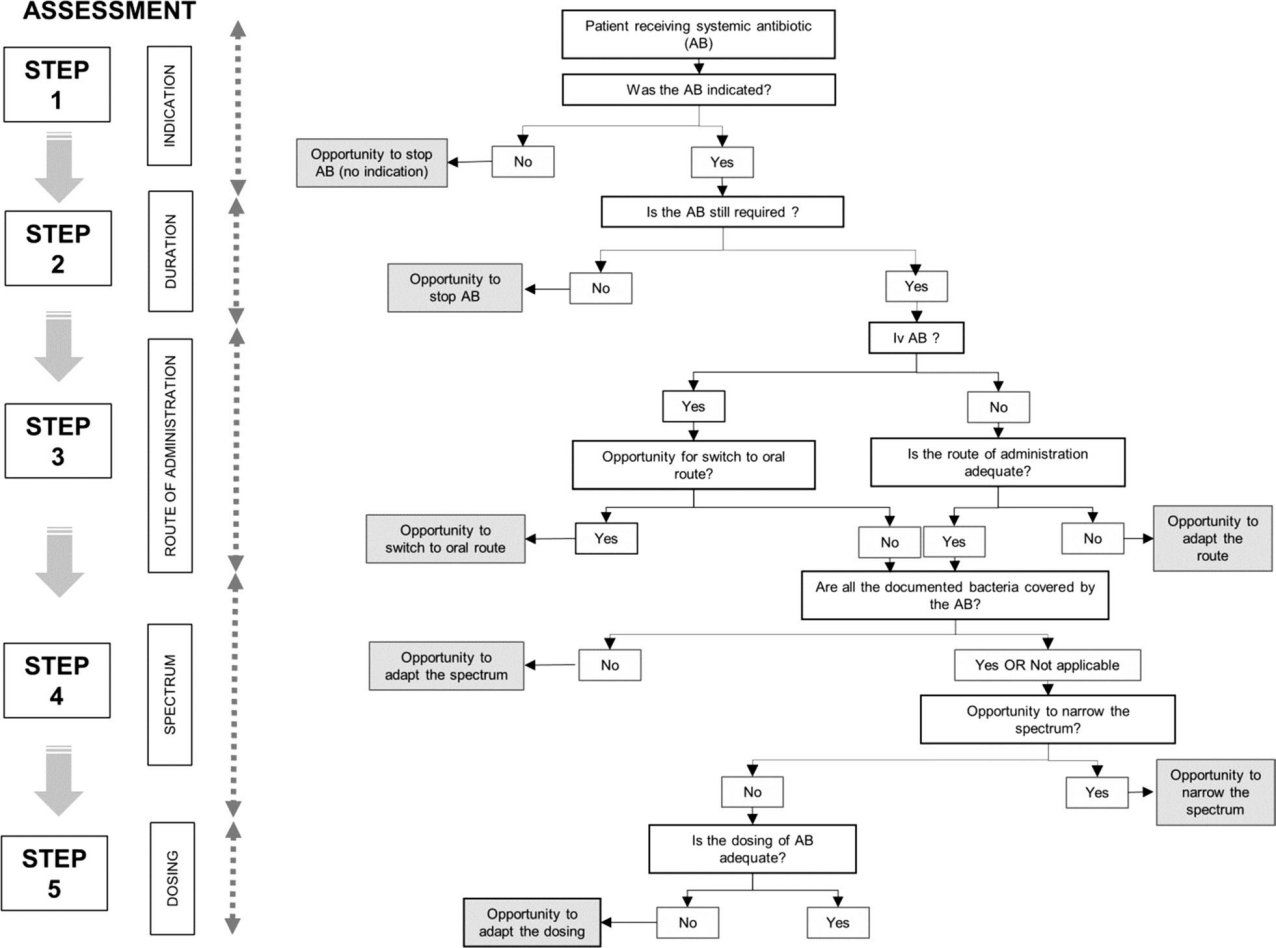
Flow chart used to assess the appropriateness of antimicrobial prescriptions

The appropriateness of antibiotic treatment—including indication, duration, administration route, spectrum and dosing—was assed according to the flow chart shown in Fig. 1 and was based on clinical data available in the patients' charts, laboratory results including microbiological documentation and radiological investigations. Assessments were made according to local guidelines and expert opinion. Institutional guidelines include for each main sites of infection the empirical treatment of first choice as well as an alternative in case of allergy, the route of administration, the usual dosages, the minimum duration of treatment and the option for oral switch. They are based on European and international evidence-based recommendations, as well as on the local epidemiology of microbial resistances. The criteria needed for oral switch were clinical improvement, in particular hemodynamic stability, availability of an oral alternative according to the microbiology results or guidelines, feasibility of oral administration and absence of an allergy to the oral alternative. A previous ID consultation performed for the current infection was recorded. Conclusions of the assessment were not communicated to the team involved in the patient management, unless the safety of the patient was affected (for example identification of a pathogen not covered by the ongoing antimicrobial therapy).

### Assessment of suitability of early discharge

The suitability of early discharge was assessed in patients receiving an antibiotic therapy by using a second flow chart following a 3-step process. Firstly, for all patients for whom the discharge was not planned on the day of the assessment, the clinical, biological and radiological evolution was evaluated. Secondly, in case of favourable evolution, the investigator checked for the absence of comorbidities requiring ongoing hospitalization, such as uncontrolled pain, nausea, inability of patients to take fluids and food by mouth or dependence on oxygen. Finally, if no comorbidity contraindicated an early discharge, independence in activities of daily living was estimated by the Katz index of Independence in Activities of Daily Living based on the nursing assessment [18]. If the patient was deemed eligible for early discharge by the investigators, the potential barriers to hospital discharge were investigated by a short interview of the physician in charge. Furthermore, the need of a flexible community facility for close medical follow-up was assessed. If the patient still needed an intravenous antimicrobial therapy, the possibility of referral to the OPAT unit was discussed. For patients on oral antibiotics the requirement for close outpatient monitoring was assessed.

Among patients considered eligible for discharge on the day of assessment, the effective date of discharge was recorded to estimate the potential reduction of hospital stay.

### Data collection and statistical analysis

Data were collected in an electronic data capture system (secuTrial).

### Ethical considerations

This point prevalence study on antibiotic appropriateness was conducted in accordance with the local ethic committee’s criteria on quality studies.

## Results

A total of 564 patient charts were reviewed over 4 months in the 31 adult somatic acute care units. In total, 182 patients (32%) received one or more antibiotics, including 62 (34%) for medical or surgical prophylaxis alone and 120 (66%) for treatment with or without an additional prophylaxis. Among patients treated with antibiotics, 90/120 (75%) were on intravenous treatment ± another route of administration and 30/120 (25%) were on oral treatment only.

Characteristics of the 120 patients receiving antimicrobials for treatment, the details of their infections and of prescribed antibiotics are summarized in Table 1. Microbiologically documented infections were mainly caused by methicillin susceptible *Staphylococcus aureus* (MSSA), enterobacteriaceae or enterococci. *Pseudomonas* spp. infections were documented in less than 2% of patients. Only one case of infection by an extended-spectrum betalactamase-producing enterobacterium was reported.

**Table 1:** Characteristics of patients receiving systemic antimicrobial treatment, n (%)

	Study population n = 120
Age (years, median, IQR)	71 (54–83)
Sex (M/F)	68/52
*Type of ward*	
Medical	48 (40.0%)
Surgical	72 (60.0%)
Comorbidities	114 (95.0%)
Heart diseases	40 (33.3%)
Diabetes	31 (25.8%)
Neurological diseases	28 (23.3%)
Respiratory diseases	27 (22.5%)
Chronic kidney disease	25 (20.8%)
Immunosuppression	53 (44.2%)
Solid organ transplant	3 (2.5%)
Solid malignancies	34 (28.3%)
Hematological malignancies	11 (9.2%)
Autoimmune diseases	9 (7.5%)
Antibiotic allergy	12 (10.0%)
Penicillin	4 (3.3%)
Cephalosporin	2 (1.7%)
Sulfamide	1 (0.8%)
*Features of infections*	
At least one nosocomial infection	43 (35.8%)
At least one episode of fever of unknown origin	5 (4.2%)
At least one clinically documented infection	43 (35.8%)
At least one microbiologically documented infection	67 (55.8%)
*Main sites of infections*	
Lungs	28 (23.3%)
Abdomen	23 (19.2%)
Bone and joints	20 (16.7%)
Urinary tract	18 (15.0%)
Surgical site	11 (9.2%)
Endocarditis	6 (5.0%)
Soft tissues	5 (4.2%)
Primary bacteremia	4 (3.3%)
Catheter	2 (1.7%)
At least one prior consultation by an ID specialist	55 (45.8%)
Prescribed antibiotics*	
Amoxicillin-clavulanate	32 (26.7%)
Piperacillin-tazobactam	25 (20.8%)
First-to third-generation cephalosporins	19 (15.8%)
Co-trimoxazole	19 (15.8%)
Vancomycin	17 (14.2%)
Other penicillins	12 (10.0%)
Carbapenems	12 (10.0%)
Quinolones	8 (6.7%)
Metronidazole	8 (6.7%)
Macrolides	3 (2.5%)

* A total of 151 antibiotic prescriptions were recorded as patients could receive combined therapies

### Assessment of antibiotic appropriateness

Regarding antibiotic prophylaxis, 25 out of 62 (40%) prescriptions were considered appropriate. Among the 37 (60%) patients who had no indication to continue antibiotic prophylaxis, 35 were hospitalized in surgical units.

Regarding antimicrobial therapies, 51 out of 120 (42%) were considered fully appropriate. Fourteen (27%) patients with appropriate treatment were hospitalized in medical units and 37 (73%) in surgical wards. The three most common infections documented among these patients were bone and joint infections (29%), abdominal infections (25%) and pneumonia (14%).

Among 69/120 (58%) patients, at least one criterion for treatment optimization was highlighted during the audit: 30 (25%) were eligible for discontinuation of antimicrobial prescriptions, 14 of whom had no indication for any antimicrobial therapy. An additional 12 patients (10%) were eligible for switching from iv to oral, 16 (13%) were eligible for spectrum adaptation, 9 (7.5%) required dosage adaptation and 2 (1.7%) were receiving inappropriate oral antibiotics. Opportunities for spectrum adaptation, specifically spectrum reduction, were identified in 17% of patients on surgical wards and in 6% on medical services. A previous ID consultation was recorded for 55/120 (46%) patients, of whom 21 (38%) could have benefited from antimicrobial therapy optimization on the day of the point assessment.

### Assessment of suitability of early discharge

**Fig. 2:**
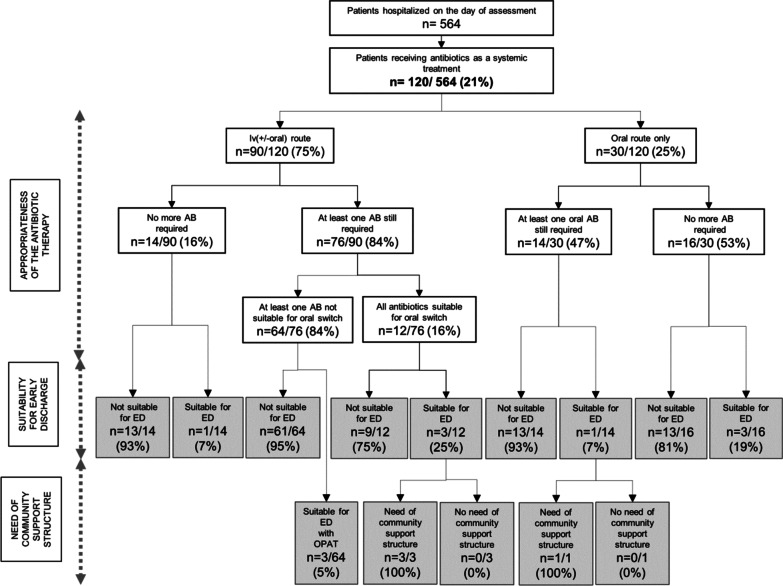
Results of the assessment of systemic antimicrobial treatments and suitability of early discharge

Discharge was already planned for the day of evaluation for 10/120 (8.3%) patients. Among the remaining 110 patients, 11 (9.2%) were considered eligible for discharge by the investigator (6 in medical units and 5 in surgical units), of whom only 4 patients (36%) were receiving appropriate treatment. Indeed, antibiotics were no longer indicated for 4 patients and switching from iv to oral route was appropriate for 3 patients. Nevertheless, the main barrier to earlier discharge mentioned by the physicians in charge was the requirement for continued clinical or biological monitoring rather than antibiotic management. Of the 11 patients eligible for early discharge two thirds could have been discharged provided that a specific outpatient structure or a close outpatient follow-up was available: 3 patients could be treated by iv route requiring support of the OPAT unit and 4 required additional close follow-up regarding infectious issues (Fig. 2). For the eleven patients eligible for an early discharge, record of effective dates of discharge showed 29 additional days in hospital (median of 2 days per patient, IQR 1–7). Among the remaining 99/110 patients, the primary reason preventing discharge according to the investigator’s assessment was the management of unstabilized comorbidities, and in particular the need for rehabilitation.

## Discussion

This is the first study combining an assessment of antimicrobial therapy appropriateness and possibility of early discharge in adult acute somatic units of a Swiss university hospital. Evaluations were conducted according to a standardized pragmatic approach in a real-life setting.

This point prevalence study showed that 32% of adult hospitalized patients received an antibiotic on a given day. In a recent Swiss national point prevalence survey on healthcare-associated infections and antimicrobial use, the prevalence of patients on one or more antimicrobials amounted similarly to 33% [19].

In our study, 60% of patients receiving an antibiotic only for prophylaxis had no longer indications to continue an antibiotic (more than 24 h following a surgical procedure), reflecting the surgeons variable adherence to guidelines about antibiotic prophylaxis durations. Based on these results, we are planning antibiotic stewardship interventions including feedback targeting this type of prescriptions. Concerning antibiotic treatments, 58% of patients presented at least one opportunity of treatment optimization despite the existence of local antibiotic prescribing resources, including access to institutional guidelines or the availability of an infectious diseases consultation, which highlights the need of more proactive antimicrobial stewardship interventions in our institution.

Interruption of antibiotic due to sufficient duration or absence of indication was the most frequent type of treatment optimization, followed by switch to oral route. These findings reflect the widespread practice of keeping antibiotics for a longer duration than necessary according to current guidelines. Lack of communication between medical teams in charge, personal habits based on old dogma, particularly regarding surgical antibiotic prophylaxis, delegation of medical decisions to junior staff, postponement of medical decisions to the next medical rounds or hesitation to modify an apparently efficacious empirical therapy, may contribute not only to unnecessary long duration of treatment, but also to prolonged broad spectrum therapies or iv administrations, increasing the risk of nosocomial infections with resistant bacteria [20]. More appropriate antibiotic use could lead to a beneficial impact on selective pressure of resistant bacteria, IV-line-related adverse events, nosocomial complications and length of hospital stay [21–23]. Hence, there is a need to proactively increase prescribers’ awareness of the importance of daily reassessment of antimicrobial therapies, regarding not only the duration, but also the opportunity to early oral switch, dosage adaptation and de-escalation.

Almost half of the patients had been evaluated by the ID team, but it is important to note that in most cases it consisted in a unique consultation several days before the point prevalence evaluation. This observation reflects the need to raise awareness about the importance of daily reassessment of antibiotic prescriptions that could be partly achieved with additional complementary ID resources dedicated to ASP interventions.

Optimization of duration and early intravenous-to-oral switch campaigns using a standardized approach have been widely applied to shorter hospital stays [23–26]. In our study, 56/120 patients (47%) of assessed patients did not require antibiotics anymore or could receive antimicrobial treatments by an oral route only. Nevertheless, eligibility for discharge could be considered for only 9.2% of evaluated patients. Proactive interventions targeting antibiotic appropriateness may not be sufficient to have an impact on length of stay [27–30]. In this study, the main reason preventing discharge at home according to the auditing investigator was the management of non-stabilized comorbidities requiring continued surgical or medical care and/or the need of transfer in a geriatric rehabilitation facility. These results should be read in perspective with the median age of included patients (71 years) and the significant proportion of these latter having comorbidities. Moreover, among the proportion of patients eligible for early discharge, two thirds would have required close ambulatory ID follow-up which sometimes cannot be provided by the general practitioner. More flexible complementary outpatient structures may be necessary to make possible and safe shorter hospital stays in patients treated with antibiotics. OPAT structures provide a real resource for antibiotic management in the ambulatory setting and could be combined with a close medical or surgical follow-up.

Our study has several limitations. Firstly, our results based on an observational point prevalence evaluation reflect practices on a given day and in a single centre. The findings can thus be influenced by local factors including institutional guidelines, hospital culture or profile of patients. Moreover, the study was conducted over 4 months during spring and seasonality can influence the antimicrobial prescription practices. Additionally, a single ID physician conducted the assessments. However, all evaluations were achieved following a systematic approach through a structured flow chart and were based on local recommendations. Antimicrobials therapies were assessed according to the information provided in the medical records. Discussions with physicians in charge could have led to additional elements that may have explained several prescriptions considered inappropriate by the auditing investigator. Finally, regarding the absence of direct feedback to prescribers, our results do not integrate a possible limitation of adhesion to propositions of optimization, which could be a limiting factor in global improvement of existing practices and has to be included in the reflections on the design of further interventions.

## Conclusion

Such point prevalence studies help hospitals to evaluate their local practices, to guide and tailor future ASP interventions and to assess resource requirement. Indeed, there is a need to prospectively study a pragmatic and global approach aiming at improving the appropriate use of antibiotics and promote early discharge, which could be applicable in routine practice. Reflections should be conducted about implementation of interventions by a dedicated team, in complementarity with existing ID consultations, to increase awareness of prescriber on the risk of antimicrobial resistance, optimize daily antibiotic prescriptions and identify prospectively suitability of early discharge as well. In addition, reflections on availability of adapted and flexible outpatient structures should be further addressed to shorten hospital stay among patients requiring antibiotics.

## Data Availability

Data were collected in an electronic data capture system (secuTrial) and are available on request to the corresponding author on reasonable request.

## Abbreviations

ASP: Antibiotic stewardship program
OPAT: Outpatient parenteral antimicrobial therapy
DDD: Defined daily doses
StAR: Swiss Antibiotic Resistance Strategy
ID: Infectious diseases
